# Metastatic gastric adenocarcinoma to the uterine cervix—a case report and review of the literature

**DOI:** 10.1186/s12957-022-02648-5

**Published:** 2022-06-03

**Authors:** Vishal Bahall, Lance De Barry, Mickhaiel Barrow, Rene Ramnarace

**Affiliations:** 1 Department of Obstetrics and Gynaecology, San Fernando General Hospital, South-West Regional Health Authority, San Fernando, Trinidad and Tobago; 2 Department of Pathology, Port of Spain General Hospital, North-West Regional Health Authority, San Fernando, Trinidad and Tobago; 3 Department of Medicine, San Fernando General Hospital, South-West Regional Health Authority, San Fernando, Trinidad and Tobago

**Keywords:** Gastric adenocarcinoma, Cervical metastasis, Oncology

## Abstract

**Background:**

Poorly differentiated diffuse-type gastric adenocarcinoma often presents at an advanced stage. While gastric cancer typically metastasizes to the liver, lung and bone, metastasis to the uterine cervix is extremely rare. To our knowledge, less than forty cases have been described in the medical literature.

**Case presentation:**

We report a case of a 47-year-old woman who presented to us with symptomatic uterine leiomyomas and subsequently underwent a successful total laparoscopic hysterectomy and bilateral salpingo-oophorectomy. The diagnosis of metastatic cancer involving the cervix was established incidentally on histopathology, which demonstrated atypical signet ring cells in the lymphovascular spaces of the cervix. Further investigations for a primary malignancy revealed a poorly differentiated diffuse-type gastric adenocarcinoma.

**Conclusion:**

Gastric cancer involving the uterine cervix is rare and associated with a poor prognosis. When presented with isolated cervical metastases, the gastrointestinal tract should be considered as a possible primary source. Due to the limited publications on this clinical entity, we expect to raise awareness and study this unique manifestation of gastric cancer by presenting our case.

## Background

Gastric cancer typically presents at an advanced stage, and up to 85% of tumours may be accompanied by lymph node metastasis at diagnosis [1]. In general, non-gynaecological malignancies rarely metastasize to the uterine cervix. Currently, less than 1% of all cervical cancers originate from an extra-genital primary, and when implicated, approximately 11.1% arise from metastatic gastric cancer [2]. Like the Krukenberg tumour of the ovary, lymphatic dissemination is regarded as the route of metastasis from primary gastric cancer to the uterine cervix [3].

The presentation of both primary cervical cancer and metastatic cancer involving the cervix is identical. Patients may be asymptomatic or present with postcoital or intermenstrual bleeding and a foul-smelling vaginal discharge [4]. Patients with advanced disease may present with pelvic or lower back pain and gastrointestinal or urinary symptoms.

Gastric cancer with metastasis to the uterine cervix carries a poor prognosis, and due to the paucity of publications on this clinical entity, there are no established treatment protocols. Hysterectomy does not improve patient outcomes; however, chemotherapy utilizing platinum-based compounds and fluoropyrimidine appear to maximize the quality of life [5].

Herein, we report a case of a 47-year-old woman who underwent a successful total laparoscopic hysterectomy and bilateral salpingo-oophorectomy for symptomatic uterine leiomyomas. Histopathology revealed an incidental finding of signet ring cells in the cervix, while immunohistochemistry suggested an adenocarcinoma of unknown primary. Further investigation for a primary malignancy with CT chest/abdomen/pelvis and upper gastrointestinal (GI) endoscopy with biopsy led to the diagnosis of poorly differentiated diffuse-type gastric adenocarcinoma.

## Case description

A 47-year-old woman presented to the gynaecology clinic with a history of menometrorrhagia and dysmenorrhea for 8 months. She denied experiencing symptomatic anaemia, postcoital bleeding, gastrointestinal and urinary symptoms and constitutional symptoms. Her past gynaecologic, obstetric and surgical history was otherwise unremarkable. She had a normal Pap smear 2 years prior. The patient had a history of gastritis which was self-treated with over-the-counter medications. She was a non-smoker and had no personal or familial history of cancer.

At the time of referral, her general physical examination was normal. Pelvic examination revealed a mobile uterus of 10 weeks size with no masses or adnexal tenderness. On speculum examination, the cervix appeared healthy and unremarkable. Pelvic ultrasonography demonstrated a solitary 6.7 cm × 4.5 cm subserosal uterine leiomyoma. There was mild left hydronephrosis with hydroureter and moderate pelvic free fluid. Blood investigations revealed a haemoglobin concentration of 10.5 g/dL and normal renal and liver function tests.

**Fig. 1:**
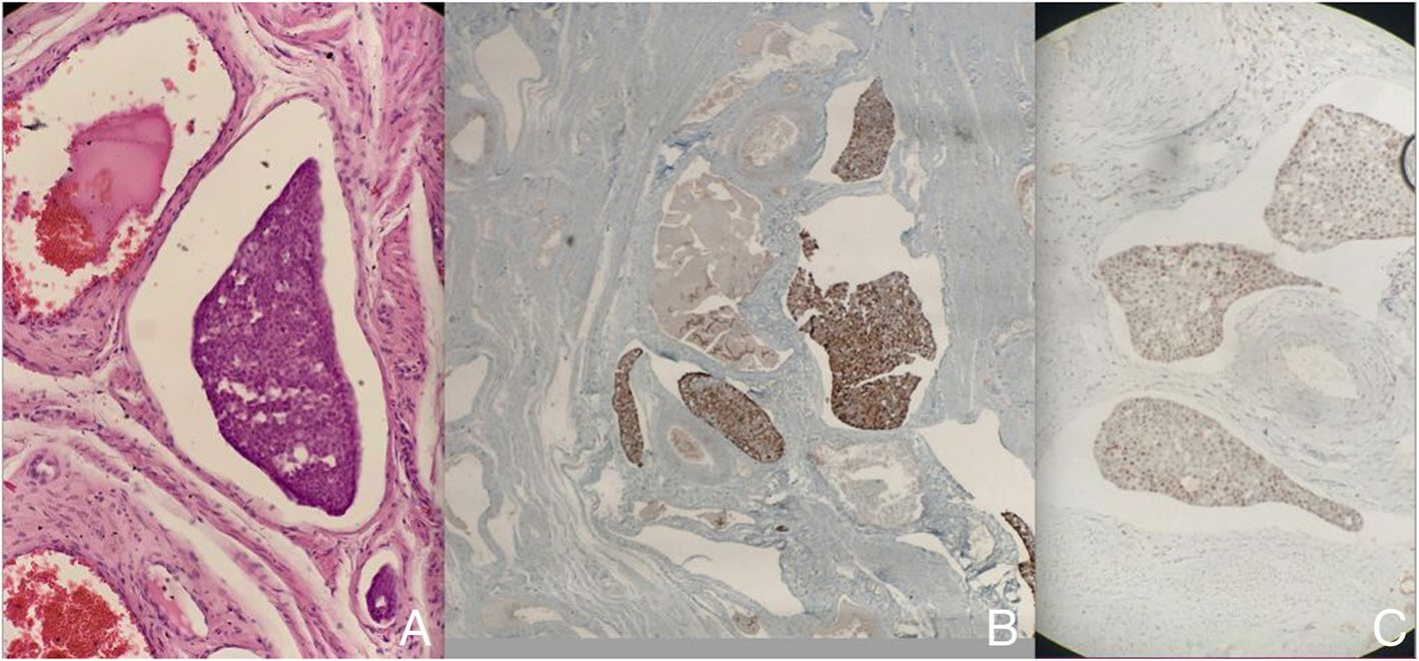
**A** Haematoxylin and eosin stain of the uterine cervix demonstrates aggregates of metastatic tumour cells within the lymphovascular spaces of the cervix. Cells display syncytial cytoplasms and round to ovoid indistinct nucleoli. **B** Immunohistochemistry demonstrates positivity for CK7. **C** Immunohistochemistry highlights a positive CDX2 stain

A tentative diagnosis of uterine leiomyoma was made, and treatment options were discussed with the patient. She opted for cystoscopy with left ureteric stenting, a total laparoscopic hysterectomy and bilateral salpingo-oophorectomy. Intraoperatively, the uterus, cervix and bilateral adnexa appeared unremarkable. The patient underwent an uneventful procedure and was discharged in satisfactory condition approximately 24 h later. Histopathology confirmed a benign uterine leiomyoma, with no evidence of malignancy. The cervix was widely sampled and there was no evidence of in situ or invasive neoplasia. However, several cervical sections revealed aggregates of atypical cells within the lymphovascular spaces of the cervix (Fig. 1A). These cells displayed moderate, syncytial cytoplasms and round to ovoid nuclei with indistinct nucleoli. Immunohistochemistry was subsequently performed, and further study of these atypical cervical cells revealed positive stains for CK7 (Fig. 1B) and CDX2 (Fig. 1C). Negative stains included CK20, CK5/6, CD45, desmin, GATA3 and PAX8. These features were highly suspicious for metastatic adenocarcinoma.

**Fig. 2:**
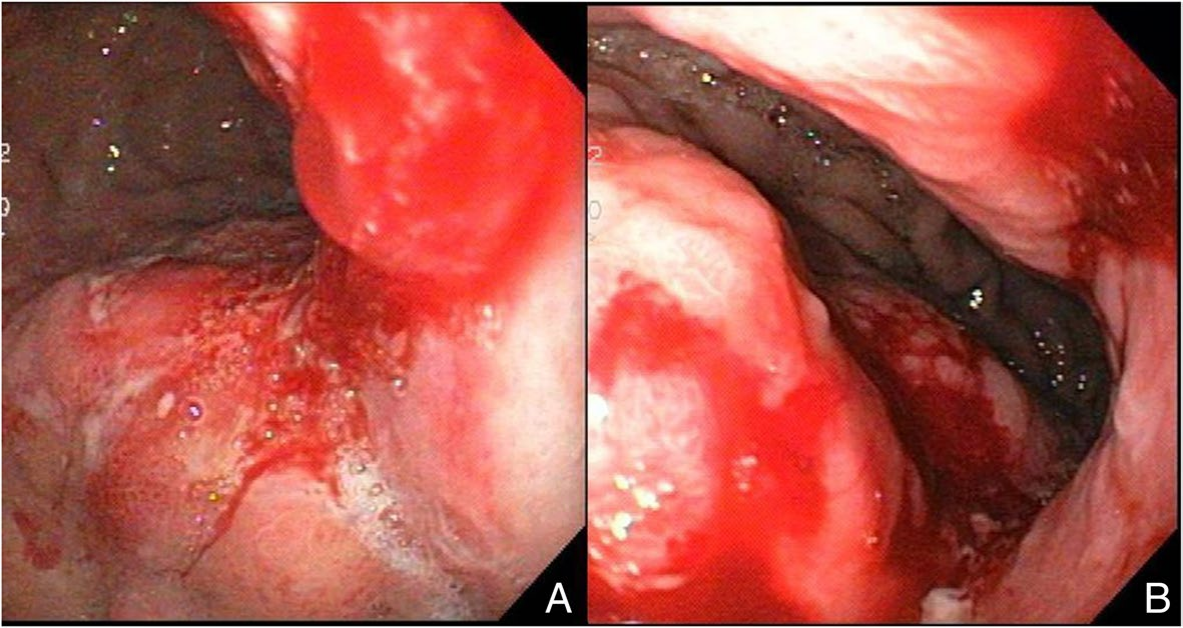
**A** Endoscopic view of the stomach demonstrates an atypical fundus with thickened, irregular folds and a diffuse gastric lesion. **B** An endoscopic view of the gastric body/lesser curvature demonstrates a friable diffuse gastric lesion with contact bleeding

A computed tomography (CT) scan of the chest, abdomen and pelvis was requested. Positron emission tomography (PET) CT scan was not available at our institution at that time. Abdominal CT demonstrated mild thickening of the gastric fundus with no associated lymphadenopathy or other features suggestive of malignancy. The intra-abdominal viscera appeared unremarkable, and the pelvic scan denoted status post hysterectomy changes. An upper gastrointestinal (GI) endoscopy was arranged, and an atypical gastric fundus with irregular thickened folds (Fig. 2) was discovered, giving the impression of a diffuse gastric lesion. The gastric cardia, antrum and pylorus appeared unremarkable. Several endoscopic biopsies were taken of the gastric fundus.

Histopathology of the gastric fundal biopsies demonstrated multiple fragments of gastric mucosa infiltrated by dissociated single cells which had high nuclear to cytoplasmic ratios and prominent nucleoli. Immunohistochemistry of gastric samples also stained positive for CK7 and CDX2. Considering the pattern of infiltration and prior cervical histopathology, it was best regarded as stage T2 N0 M1 poorly differentiated diffuse-type gastric adenocarcinoma that metastasized to the uterine cervix. The patient’s case was subsequently discussed at the multidisciplinary team (MDT) meeting, and a decision was made for the patient to undergo chemotherapy due to the presence of metastatic disease in the cervix. Surgery for the primary gastric cancer was not considered. The patient was referred to the Medical Oncology team for further management. However, she eventually decided to stop treatment due to the side effects of chemotherapy and the progression of the disease. The patient succumbed to her disease a few months later.

## Discussion

Gastric cancer is the fifth most common cancer among women and the fourth leading cause of cancer-related death worldwide [6]. The most prevalent histopathologic subtype of gastric cancer is adenocarcinoma which is subdivided into the intestinal and diffuse types according to the Lauren classification [1]. Additional subtypes of gastric cancer include signet ring cell carcinoma, adenosquamous carcinoma and squamous cell carcinoma [7]. Gastric cancer often metastasizes to the liver (48%), peritoneum (32%), lung (15%) and bone (12%) [8]. Metastatic gastric cancer presenting in the uterine cervix is extremely rare.

In general, metastasis to the female genital tract from an extrapelvic malignancy is highly unusual. Regardless of the location of primary cancer, metastasis commonly affects the ovaries and vagina [9]. The cervix is less commonly affected compared to the uterine corpus [9, 10]. Moreover, less than 1% of all cervical malignancies originate from a non-gynaecological metastatic primary. These metastatic primary cancers originate from the breast (42.9%), colon (17.5%), stomach (11.1%), pancreas (11.1%), gallbladder (4.8%), cutaneous melanoma (3.2%), urinary bladder (3.2%) and thyroid (1.6%) [2]. In 1999, one study that examined 40 cases of primary extra-genital malignancies with metastasis to the cervix found that the most common source of primary malignancy originated from breast cancer and gastric cancer [11].

Non-gynaecological cancers may metastasize to the cervix according to haematogenous dissemination, retrograde lymphatic spread and transperitoneal seeding [10, 11]. Wallach and Edberg proposed several reasons for the mode of metastasis to the uterine cervix. These include the small size of the target area, the limited blood and lymphatic supply of the cervix and the unfavourable conditions for the growth of a tumour in the fibromuscular stroma [11]. Similar to the Krukenberg tumour of the ovary, it is postulated that lymphatic dissemination is responsible for metastasis from gastric primary cancer to the cervix [11].

The lymphatic vessels of the cervix drain in a circumferential pattern [3, 10]. Metastasis by this route may only occur if tumour emboli obstruct distant lymphatic channels [12]. In postmenopausal women, the high density of fibrous tissue and decreased blood supply of the cervix provide conditions that are unsuitable for the growth and survival of metastatic tumour cells [3, 12]. Therefore, patients suffering from metastatic cancer to the cervix are typically young and premenopausal [11, 13]. In the case presented, histopathology demonstrated aggregates of metastatic cells in the lymphovascular spaces of the cervix and thus correlated with clinical evidence for the mode of metastasis of extra-pelvic primary malignancies to the uterine cervix.

The presentation of primary cervical cancer and metastatic cancer to the cervix is identical [13]. Patients commonly report abnormal vaginal bleeding, post-coital bleeding, intermenstrual bleeding and a malodorous vaginal discharge [14]. Patients with advanced disease may manifest gastrointestinal or urinary symptoms and pelvic or lower back pain [15]. In the case of metastasis to the cervix, symptoms of primary cancer may or may not be evident. On routine screening, early cervical disease is usually asymptomatic and detected by an abnormal cervical smear [16]. Rarely, abnormal glandular cells are seen on cervical smears which may be suggestive of ectopic tumour cells [10, 11]. According to the American Society for Colposcopy and Cervical Pathology (ASCCP) 2019 guidelines, recommendations for cervical smear, colposcopy, treatment or surveillance are based on a patient’s risk of CIN III (cervical intraepithelial neoplasia) as determined by a combination of current results and history of past test results [17]. For women between 30 and 65 years of age, cervical cytology is recommended every 3 years or every 5 years if HPV co-testing is negative [17]. Patients who have consistently tested negative on cervical cytology and/or HPV co-testing can undergo hysterectomy if their last cervical smear was within 3 years of testing [17]. According to these recommendations by the ASCCP, our patient did not undergo cervical cytology immediately prior to hysterectomy since she consistently tested negative on routine Pap smears, and her last test was within the recommended 3-year period.

Likewise, the pelvic examination findings in patients with metastatic cervical disease often resemble that of primary cervical cancer [14]. Such findings include a friable bleeding cervical lesion or mass with possible invasion into the upper vagina. The cervix may also appear enlarged and stony hard [11, 14]. However, Imachi reported that in up to 50% of patients, the cervix was found to be normal on pelvic examination [10]. This highlights the fact that cytologic and histological assessments of the cervix are necessary for the diagnosis of metastatic adenocarcinoma to the cervix.

In the workup for primary cancer, immunohistochemistry (IHC) guides the identification of carcinomas of unknown primary sites, tumour classification, behaviour and prognosis [18]. An initial immunohistochemistry profile performed on a suspicious tissue sample covers a broad spectrum of cytokeratins such as CK7 and CK20 followed by organ-specific markers like PAX8, CDX-2 and GATA3 [19]. Both intestinal and diffuse-type gastric adenocarcinoma show variable expression for CK7 and CK20 [18, 20]. Specifically, CDX-2 is identified in over 70% of diffuse-type gastric adenocarcinoma [19]. Findings in our patient reflected evidence in the current literature as both cervical and gastric immunohistochemistry demonstrated positivity for CK7 and CDX-2.

The prognosis in patients with gastric metastasis to the cervix is poor, and unfortunately, a total hysterectomy does not improve clinical outcomes [21]. Although there are no established treatment protocols for patients diagnosed with metastatic gastric cancer to the uterine cervix, chemotherapy utilizing platinum-based compounds combined with fluoropyrimidine appears to maximize the quality of life and prolong patient survival [5, 9]. Currently, reports that indicate successful outcomes with radiotherapy are scarce, and there are no standardized protocols available. According to Yamamoto et al., the median survival from the time of discovery of cervical metastasis is 4 months [22].

In conclusion, this case highlights a rare metastatic pattern of poorly differentiated diffuse-type gastric adenocarcinoma in a 47-year-old woman. The incidental histopathological finding of atypical cells in the lymphovascular spaces of the cervix following surgery for benign uterine leiomyoma led to the discovery of primary gastric cancer. For the unsuspecting gynaecologist, isolated cervical metastasis should be considered part of the differential diagnosis when atypical cells are identified in the cervix and the gastrointestinal tract should be considered a possible primary source. Furthermore, immunohistochemistry has a vital role in identifying the primary tumour site. Until treatment protocols are established, chemotherapy utilizing fluoropyrimidine and platinum-based compounds may maximize the quality of life in these patients.

## Data Availability

Not applicable.

## Abbreviations

CT: Computed tomography
GI: Gastrointestinal
GIST: Gastrointestinal stromal tumour
MDT: Multi-disciplinary team meeting
